# Doxorubicin and resveratrol co-delivery nanoparticle to overcome doxorubicin resistance

**DOI:** 10.1038/srep35267

**Published:** 2016-10-12

**Authors:** Yuan Zhao, Meng-lei Huan, Miao Liu, Ying Cheng, Yang Sun, Han Cui, Dao-zhou Liu, Qi-bing Mei, Si-yuan Zhou

**Affiliations:** 1 Department of Pharmaceutics, School of Pharmacy, Fourth Military Medical University, Xi’an, 710032, China; 2 Key laboratory of gastrointestinal pharmacology of Chinese medicine, Fourth Military Medical University, Xi’an, 710032, China

## Abstract

With the extensive application of doxorubicin (DOX), DOX resistance has become one of the main obstacles to the effective treatment of breast cancer. In this paper, DOX and resveratrol (RES) were co-encapsulated in a modified PLGA nanoparticle (NPS) to overcome the DOX resistance. CLSM results indicated that DOX and RES were simultaneously delivered into the nucleus of DOX-resistant human breast cancer cells by DOX/RES-loaded NPS. Consequently, DOX/RES-loaded NPS showed significant cytotoxicity on MDA-MB-231/ADR cells and MCF-7/ADR cells. Furthermore, DOX/RES-loaded NPS could overcome DOX resistance by inhibiting the expression of drug resistance-related protein such as P-gp, MRP-1 and BCRP, and induce apoptosis through down-regulating the expression of NF-κB and BCL-2. In tumor-bearing mice, DOX/RES-loaded NPS mainly delivered DOX and RES to tumor tissue. Compared with free DOX, DOX/RES-loaded NPS significantly inhibited the DOX-resistant tumor growth in tumor-bearing mice without causing significant systemic toxicity. In a word, DOX/RES-loaded NPS could overcome the DOX resistance and had the potential in the treatment of DOX-resistant breast cancer.

Doxorubicin (DOX), with the advantages of exact curative effects and low price, was used as the first-line chemotherapeutic drug to treat breast cancer for a long time. But the serious toxicity to normal tissues such as cardiotoxicity and development of DOX resistance limit the clinical application of DOX. One of the important mechanisms of DOX resistance is considered as the over-expression of ATP-binding cassette (ABC)^1^. ATP-binding cassette is a protein super-family which can actively pump a broad variety of endogenous molecules and xenobiotics out of cells from low concentration site to high concentration site^2^. P-glycoprotein (P-gp), multidrug resistance protein 1 (MRP-1) and breast cancer resistance protein (BCRP) are the primary ABC protein, which is related to DOX resistance^1^,^3^,^4^.

In recent years, numerous strategies were proposed to reverse DOX resistance. An important method to overcome DOX resistance is to use two or more chemotherapeutic drugs with different anti-tumor mechanisms^5^. However, the pharmacokinetic properties of different drugs are vastly different. Therefore, just using two or more free chemotherapeutic drugs without controlling their delivery and release characteristic can not effectively overcome DOX resistance or enhance the anticancer effects of the drugs^6^,^7^. The nanoparticles that co-delivered anticancer drug and ABC transporter inhibitor showed great improvement in the efficacy of chemotherapeutic drug^8^,^9^,^10^.

Many researchers have found that resveratrol (RES) can inhibit P-gp activity and subsequently increase the intracellular accumulation of the chemotherapeutic drug. Eventually, RES improve the sensitivity of tumor cells to the chemotherapeutic drug^11^,^12^,^13^,^14^. Besides, Shukla Y. *et al*. found that RES increased the chemosensitivity of DOX to breast cancer cells through inducing apoptosis in tumor cells and inhibiting proliferation and invasion of tumor cells^15^. Furthermore, RES can reduce cardiac toxicity and fibrosis that induced by DOX^16^,^17^. However, the metabolism of RES is extremely rapid and extensive, and its plasma half-life is only about 9 min in human^18^. Moreover, the oral bioavailability of RES is nearly zero due to its poor water solubility and significant first-pass effect^18^,^19^,^20^. On the other side, the half life of free DOX is about 30~40 h in human^21^,^22^. Therefore, just using the mixture of free DOX and free RES without controlling their pharmacokinetic characteristic can not overcome DOX resistance and enhance the anticancer effects of DOX.

In this study, DOX and RES were co-encapsulated in poly(lactic-co-glycolic acid) (PLGA) based nanoparticles to prolong the half-life of DOX and RES, increase the concentration of DOX and RES in tumor tissue, overcome DOX resistance, and reduce the toxicity of DOX in healthy organs. Also, Sigma receptor was reported to over-express in a variety of human tumor cells including, breast cancer^23^,^24^. Thus, anisamide (AA), a high-affinity ligand for Sigma receptors, was used to increase the selectivity of PLGA nanoparticles to tumor cells. The anti-tumor activity of DOX/RES-loaded nanoparticles on DOX-resistant tumor and its molecular mechanisms were deeply investigated *in vitro* and *in vivo*.

## Materials and Methods

### Materials

The p-anisoyl chloride, 4-acetylbenzoic acid, dimethylaminopyridine (DMAP), 1-ethyl-(3-dimethylaminopropyl) carbodiimide hydrochloride (EDC), N-hydroxysuccinimide (NHS), poly(lactic-co-glycolic acid) (PLGA, lactic acid/glycolic acid molar ratio is 75/25, and weight average molecular weight is 40–75 kDa) was bought from EVONIK Industries (Recklinghausen, German). Triethylamine (TEA), trifluoroacetic acid (TFA), cholesterol and tertbutyl carbazate (Boc-NH-NH_2_) were purchased from Sinopharm Chemical Reagent Co., Ltd (Shanghai, China). NH_2_-PEG-NH_2_ (MW 2000) was bought from Shanghai Yare Biotech Inc. (Shanghai, China). 3-(4,5-dimethylthiazol-2-yl)-2,5-diphenyltetrazolium bromide (MTT) was obtained from Sigma-Aldrich company (St. Louis, MO, USA). Resveratrol was supplied by the Xi’an Xinrui Biological Engineering Technology Company (Xi’an, China). DOX·HCl was got from Hisun Pharmaceutical Company (Zhejiang, China). The cell culture medium RPMI-1640 and 4′,6-diamidino-2-phenylindole (DAPI) were purchased from Invitrogen Technologies Company (Carlsbad, USA). The anti-MRP-1 antibody, anti-BCRP antibody, anti-p-glycoprotein antibody, anti-NF-κB p65 antibody, anti-MRP1 antibody and anti-β-actin antibody were purchased from Abcam (Cambridge Science Park, British). The anti-Bax antibody, anti-BCL-2 antibody, anti-CyclinA antibody, anti-cyclin D1 antibody, anti-CDK2 antibody, anti-CDK4 antibody, anti-rabbit IgG H&L (HRP) and Goat anti-Mouse IgG H&L (HRP) were bought from Proteintech (Chicago, USA).

### Animal and cancer cell line

The 5-week-old female BALB/c nude mice (the body weight was about 21~23 g) were got from Experimental Animal Center of Fourth Military Medical University.

The experimental protocols were approved by the Animal Care and Use Committee of Fourth Military Medical University (protocol number: 15004), and all methods were performed in accordance with the relevant ethical guidelines and regulations of Fourth Military Medical University.

MCF-7/ADR cell (DOX-resistant estrogen receptor positive human breast adenocarcinoma cell) was supplied by Institute of Biochemistry and Cell Biology, Chinese Academy of Science (Shanghai, China). MBA-MD-231/ADR cell (DOX-resistant estrogen receptor negative human breast adenocarcinoma cell) was induced by our lab. DOX (2 μmol/L) was added in the medium and cultured with MBA-MD-231/ADR cells and MCF-7/ADR for 24 h once a week to maintain the DOX-resistant property^25^.

### Synthesis of CHO-hyd-PEG-AA

CHO-hyd-PEG-AA was synthesized according to Supplementary Figure 1.

The cholesterol (5.0 g, 13 mmol) was dissolved in dichloromethane (20 mL), and succinic anhydride (1.9 g, 19 mmol), DMAP (771.1 mg, 6.4 mmol) and TEA (910.7 mg, 9 mmol) were successively added into the dichloromethane solution. The mixture was stirred at room temperature for 24 h. Then the cholesterol-hemisuccinate (CHO-suc) was separated and purified by silica gel column (petroleum ether/ethyl acetate, v/v = 1/2). Then, the CHO-suc (486.4 mg, 1.0 mmol), EDC (191.0 mg, 1.0 mmol) and NHS (115.1 mg, 1.0 mmol) were dissolved in dichloromethane (6 mL), and the reaction mixture was stirred for 6 h at room temperature. Then Boc-NH-NH_2_ (132.2 mg, 1 mmol) and TEA (0.1 mL) were added to the reaction mixture, and the reaction mixture was stirred for 12 h at room temperature. The dichloromethane was evaporated, and cholesterol-succinic-NH-NH-Boc was purified by silica gel column (petroleum ether/ethyl acetate, v/v = 2/1). Finally, cholesterol-succinic-NH-NH-Boc was dissolved in dichloromethane (2 mL), and the catalytic amount of TFA (0.5 mL) was added. The mixture was stirred for 2 h at room temperature. The reaction mixture was washed with water (20 mL) for three times. The organic phase was collected and evaporated. The residue was purified by silica gel column (petroleum ether/ethyl acetate, v/v = 2/1) to get cholesterol-succinic-NH-NH_2_ (CHO-NH-NH_2_).

AA-PEG-4-acetylbenzoic acid was synthesized by using p-anisoyl chloride, PEG and 4-acetylbenzoic acid^26^. The AA-PEG-4-acetylbenzoic acid (150.3 mg, 0.03 mmol) and CHO-NH-NH_2_ (30.2 mg, 0.06 mmol) were dissolved in 5 mL dichloromethane and the catalytic amount of TFA (0.2 mL) was added, and the mixture was stirred for 8 h at room temperature. After that, the reaction solvent was evaporated, and the residue was dissolved in water (20 mL). The solution was removed into dialysis tube, and it was dialyzed in water for 48 h. The solution in dialysis tube was freeze-dried to get CHO-hyd-PEG-AA.

### Preparation and characterization of DOX/RES-loaded PLGA nanoparticles (NPS)

DOX/RES-loaded NPS was prepared by using emulsion-solvent evaporation method. Briefly, 10 mg PLGA and 5 mg CHO-hyd-PEG-AA were dissolved in dichloromethane (3 mL), 8 mg RES was dissolved in ethanol (0.5 mL), and 3 mg DOX·HCl was dissolved in distilled water (6 mL). The above solution was mixed and emulsified by using ultrasound for 3 min at 0 °C. After that, 0.3% PVA (10 mL) was added into the mixture solution and further emulsified by using ultrasound for 3 min at 0 °C. Finally, the mixture solution was stirred at room temperature for 4 h to remove dichloromethane. After centrifugation, the precipitation was re-suspended in water, and then frozen-dried to obtain DOX/RES-loaded NPS powder. The zeta potential and size distribution of DOX/RES-loaded NPS were detected at room temperature by zeta-potential & particle size analyzer (Beckman Coulter, Miami, USA). The morphology of DOX/RES-loaded NPS was observed by transmission electron microscopy (TEM, JEOL, Japan). The drug loading (DL) and drug release characteristic from DOX/RES-loaded NPS *in vitro* were determined by using 970 CRT fluorescence spectrophotometer (Shanghai Precision & Scientific Instrument Co. Ltd, Shanghai, China).

### Cytotoxicity of DOX/RES-loaded NPS

The store solution of RES was prepared by using dimethylsulfoxide (DMSO). The final RES solution used to treat cell contained less than 0.3% DMSO. DOX·HCl and DOX/RES-loaded NPS was directly dissolved with cell culture medium.

MDA-MB-231/ADR cells and MCF-7/ADR cells were seeded in 96-well plates at a density of 5 × 10^4 ^cells/mL and cultured for 24 h. The culture medium was replaced by fresh RPMI 1640 (200 μL) or fresh RPMI 1640 containing blank NPS, DOX/RES-loaded NPS, free DOX, free RES and MIX (a mixture of free DOX and free RES), and the cells were cultured for 48 h. Then, 20 μL MTT solution (5 mg/mL) was added into each well and incubated the cells for another 4 h. Finally, cell culture medium was replaced by 200 μL DMSO, and the absorbance (OD) of DMSO solution was detected by using the microplate reader (Bio-Rad Laboratories, Inc. Richmond, California, USA) at 490 nm. Cell viability was calculated by the following formula:


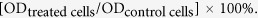


### Caspase 3 activity induced by DOX/RES-loaded NPS

To evaluate the effect of DOX/RES-loaded NPS on caspase 3 activity in MDA-MB-231/ADR cells and MCF-7/ADR cells, cells were seeded in 6-well plates at a density of 5 × 10^5 ^cells/mL and incubated for 24 h. Then the cells were exposed to DOX/RES-loaded NPS, free DOX, free RES and MIX (the equivalent concentration of RES was 60 μg/mL and 90 μg/mL, the equivalent concentration of DOX was 20 μg/mL and 30 μg/mL, respectively) for 24 h. Caspase 3 activity assay kit (Beyotime Biotechnology, China) was used to detect caspase 3 activity in MDA-MB-231/ADR cells and MCF-7/ADR cells.

### Cellular uptake of DOX/RES-loaded NPS

RES shows green fluorescence whereas DOX shows red fluorescence. Confocal laser scanning microscopy was used to evaluate cellular uptake of DOX/RES-loaded NPS. MDA-MB-231/ADR cells and MCF-7/ADR cells were respectively seeded in 24-well plates with a cover glass in each well (5 × 10^4 ^cells/mL) and incubated for 24 h. Then the cells were incubated with DOX/RES-loaded NPS, free DOX, free RES and MIX (the equivalent concentration of RES and DOX was 30 μg/mL and 10 μg/mL, respectively) for 4 h. The culture medium was then removed, and the cells were slightly washed with PBS for 3 times. The cells were exposed to 500 μL DAPI (100 μg/mL, Invitrogen Technologies Company, Carlsbad, USA) for 15 min. After DAPI medium had been removed, the cells were slightly washed 3 times with PBS. After that, the cells were fixed for 15 min at room temperature by using 4% paraformaldehyde. Finally, the glycerol was dropped on slides to seal cell samples. The Olympus FV10-ASW (Tokyo, Japan) was used to detect the localization of RES green fluorescence and DOX red fluorescence in the cell, and Image Jv1.47 software packages were used to quantitatively analyze the relative fluorescence intensity in the cell.

### Cell cycle analysis

Flow cytometry was used to evaluate the effect of DOX/RES-loaded NPS on the cell cycle. Briefly, MCF-7/ADR cells and MDA-MB-231/ADR cells were seeded at a density of 5 × 10^5 ^cells/mL in 6-well plates and incubated for 12 h. Then the cells were culture with the serum-free RPMI 1640 (200 μL) for 12 h to synchronize the cells. After that, the culture medium was replaced by fresh RPMI 1640 (200 μL) or fresh RPMI 1640 containing DOX/RES-loaded NPS, free DOX, free RES and MIX (the equivalent concentration of RES and DOX was 60 μg/mL and 20 μg/mL, respectively), and the cells were incubated for 24 h. Then the cells were harvested and suspended in 75% ethanol at 4 °C for 12 h. The cells were collected by centrifugation. Finally, the cells were incubated with mixture of Triton X-100 (0.1%,v/v), RNAseA (1 μg/mL) and propidium iodide (0.2 mg/mL) at 37 °C for 1 h. Cell cycle was detected by flow cytometry (BD FACSAria, USA).

In addition, after being treated with DOX/RES-loaded NPS for 24 h, cells were harvested and lyzed with RIPA lysis buffer for 15 min. The supernatant was collected after the cells lysis was centrifugated (16000 × g, 4 °C) for 15 min, and the protein concentration was quantified by Bradford reagent. The cell cycle-related proteins were analyzed by western blot.

### Drug resistance-related protein and apoptosis-related protein expression in tumor cells

MCF-7/ADR cells and MDA-MB-231/ADR cells were seeded at a density of 5 × 10^5 ^cells/mL in 6-well plates and incubated for 24 h. The culture medium was replaced by fresh RPMI 1640 (200 μL) or fresh RPMI 1640 containing DOX/RES-loaded NPS, free DOX, free RES and MIX (the equivalent concentration of RES and DOX was 60 μg/mL and 20 μg/mL, respectively), and the cells were incubated for 24 h. The expression of drug resistance-related proteins such as MRP-1, BCRP and p-gp, and apoptosis-related proteins such as NF-κB, Bax and BCL-2 were measured by western blot method.

### *In vivo* antitumor activity

The 5-week-old female BALB/c nude mice were subcutaneously implanted with MDA-MB-231/ADR cells (1 × 10^7 ^cells/0.1 mL/animal) in rear right flank. As the tumor mass grew to a visible size (about 170 mm^3^), the mice were randomly divided into 6 groups (5 mice/each group). The tumor-bearing mice were intravenously injected with DOX/RES-loaded NPS (equivalent DOX dose was 5 and 10 mg/kg, respectively), DOX (5 and 10 mg/kg), RES (30 mg/kg) and normal saline every 5^th^ day. The mice were observed and weighted every day. Tumor growth was monitored by measuring the perpendicular diameter of the tumor using a caliper. The tumor volume was calculated according to the following formula: tumor volume (mm^3^) = 0.5 × length × width^2^. The level of drug resistance-related protein and apoptosis-related protein in tumor tissue were measured by western blot method at the end of the experiment.

### *In vivo* drug distribution

The tumor-bearing mice were intravenously injected with DOX/RES-loaded NPS (equivalent DOX and RES dose was 10 mg/kg and 30 mg/kg, respectively), DOX (10 mg/kg) and RES (30 mg/kg) by tail vein. After drug was administered for 12, 24 and 48 h, the mice were sacrificed. Tumor tissues and organs were excised and rinsed in PBS (pH 7.4), and Caliper IVIS Lumina II *in-vivo* image system (Caliper Life Science, USA) was used to evaluate the time-dependent drug distribution in tumor tissues and various organs. Living Image 4.2 software was used to quantitatively analyze the fluorescence intensity in tumor tissues and organs. At the same time, the tumor tissues and organs were cut to 5 μm thickness sections, and sections were stained with DAPI (100 μg/mL). Fluorescence microscopy (DS-5M-ui80i, Nikon Corporation, Japan) was used to observe RES green fluorescence and DOX red fluorescence in the section of tumor tissues and organs.

### Histopathological analysis

At the end of the *in vivo* antitumor experiment, the tumor tissues and organs were cut to 5 μm thickness sections, and the sections were stained with H&E (hematoxylin and eosin) to observe the histomorphological changes.

### Statistical Analysis

Data were expressed as the mean ± standard deviation. The comparison of each group was assessed by using one-way ANOVA analysis of Origin8 software (SPSS Inc., Chicago, IL, USA). The level of significance was indicated by *p < 0.05, **p < 0.01.

## Results

### Characterization of CHO-hyd-PEG-AA

The ^1^HNMR spectrum and infrared spectrum of CHO-hyd-PEG-AA are presented in Supplementary Figures 2 and Fig. 3. The AA in CHO-hyd-PEG-AA was confirmed by the signals at peak a, b, c, d, f (δ = 2.38, δ = 8.2, δ = 7.74, δ = 6.19, δ = 1.46). The signal at peak g (δ = 3.6 ppm) confirmed the PEG backbone in the conjugate. The signal at peak h (δ = 5.35 ppm) confirmed the CHO backbone in the conjugate. The characteristic absorbance for PEG was verified in 1113/cm (C-O-C) and about 774/cm (C-H). The absorption at about 1661/cm was attributable to C=N of the hydrazone bond.

### Characterization of NPS

**Figure 1:**
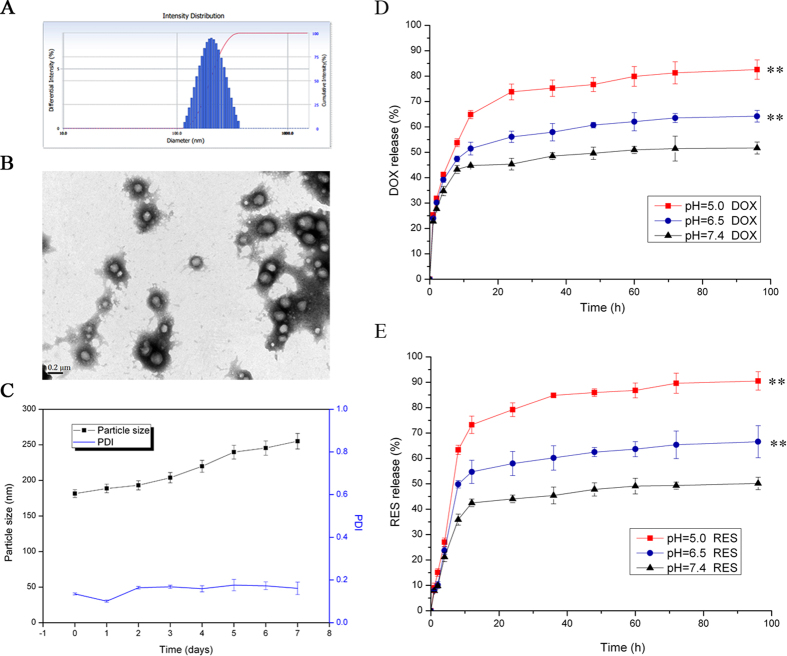
Characteristics of DOX-RES-loaded nanoparticles (NPS). Panel (A) is particle size distribution of NPS measured by dynamic light scattering. Panel (B) is TEM image of NPS. Panel (C) are the changes of particle size and particle distribution index of NPS in PBS for 7 days at 37 °C, measured by dynamic light scattering. Panels (D,E) are *in vitro* DOX and RES release curve from DOX/RES-loaded NPS in different PBS (pH 5.0, pH6.5 and pH 7.4) at 37 °C. Data are mean ± SD. n = 3, **p < 0.01, vs pH 7.4 at 96 h. All experiments were tested for three times.

The particle size distribution, TEM images, and stability in water of DOX/RES-loaded NPS are showed in Fig. 1A–C, respectively. The particle size of DOX/RES-loaded NPS was 170 nm with the polydispersity index (PDI) of 0.14. The zeta potential of DOX/RES-loaded NPS was −15.8 ± 0.8 mV. The TEM image showed that DOX/RES-loaded NPS was generally spherical in shape and kept good dispersity. The drug loading of NPS was (4.6 ± 1.4)% for DOX and (15.5 ± 5.3)% for RES. The release rate of DOX and RES from the DOX/RES-loaded NPS in different pH medium is showed in Fig. 1D,E. DOX/RES-loaded NPS released out 44% of loaded DOX and 42% of loaded RES in 24 h in pH 7.4 medium. However, in pH 5.0 medium, DOX/RES-loaded NPS released out 73% of loaded DOX and 81% of loaded RES in 24 h, respectively. This result implied that DOX and RES from NPS exhibited pH-dependent manner.

### Cytotoxicity of DOX/RES-loaded NPS

**Figure 2:**
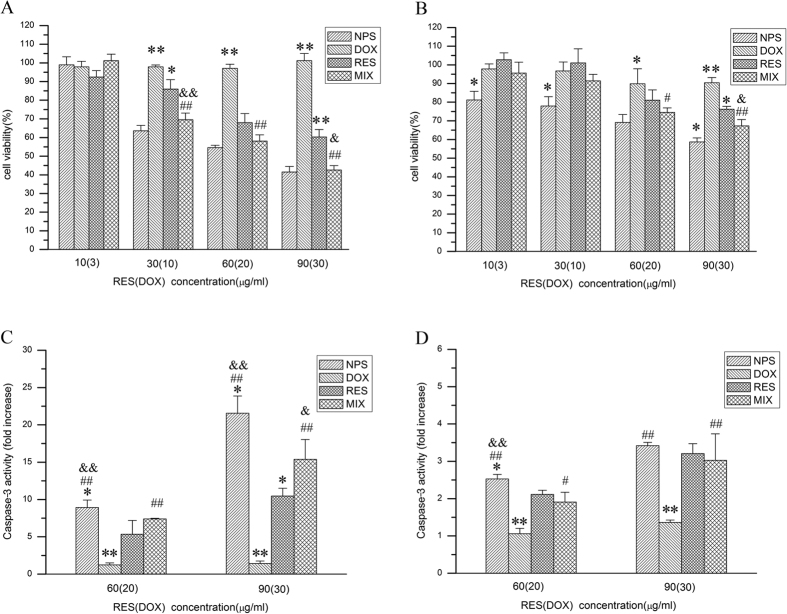
Cytotoxicity of free doxorubicin (DOX), free resveratrol (RES), mixture of free DOX and free RES (MIX) and DOX/RES-loaded nanoparticles (NPS) on the MDA-MB-231/ADR cells (panel A) and MCF-7/ADR cells (panel B) in 48 h at 37 °C. The caspase-3 activity in MDA-MB-231/ADR cells (panel C) and MCF-7/ADR cells (panel D) after cells were incubated with free DOX, free RES, MIX and NPS for 24 h. Data are mean ± SD. n = 3. *p < 0.05, **p < 0.01, vs same concentration of MIX. ^#^p < 0.05, ^##^p < 0.01, vs same concentration of free DOX. ^&^p < 0.05, ^&&^p < 0.01, vs same concentration of RES.

As showed in Supplementary Figure 4A,B, blank NPS did not exhibit significant cytotoxicity both on MDA-MB-231/ADR cells and MCF-7/ADR cells as dose ranged from 50 to 800 μg/mL. The cytotoxicity of DOX/RES-loaded NPS on MDA-MB-231/ADR cells and MCF-7/ADR cells are showed in Fig. 2A,B, respectively. Free DOX showed no obvious cytotoxicity on MDA-MB-231/ADR cells and MCF-7/ADR cells. However, free RES inhibited the proliferation of MDA-MB-231/ADR cells and MCF-7/ADR cells in dose-dependent manner. Compared with free DOX and free RES, the mixture of free DOX and free RES showed significantly higher cytotoxicity on MDA-MB-231/ADR cells and MCF-7/ADR cells. Besides, DOX/RES-loaded NPS exhibited the highest cytotoxicity on MDA-MB-231/ADR cells and MCF-7/ADR cells in four groups. Compared with MCF-7/ADR cells, MDA-MB-231/ADR cells was more sensitive to DOX/RES-loaded NPS.

### The effect of DOX/RES-loaded NPS on caspase-3 activity in MDA-MB-231/ADR cells and MCF-7/ADR cells

The effect of DOX/RES-loaded NPS on the caspase-3 activity in MDA-MB-231/ADR cells and MCF-7/ADR cells are showed in Fig. 2C,D, respectively. The caspase-3 activity in DOX/RES-loaded NPS-treated cells increased in dose-dependent manner. Besides, DOX/RES-loaded NPS and mixture of free DOX and RES induced a greater activity of caspase-3 in MDA-MB-231/ADR cells than in MCF-7/ADR cells. The above results were well consistent with the cytotoxicity of DOX/RES-loaded NPS on MDA-MB-231/ADR cells and MCF-7/ADR cells.

### Cellular uptake and intracellular localization of DOX and RES delivered by NPS

**Figure 3:**
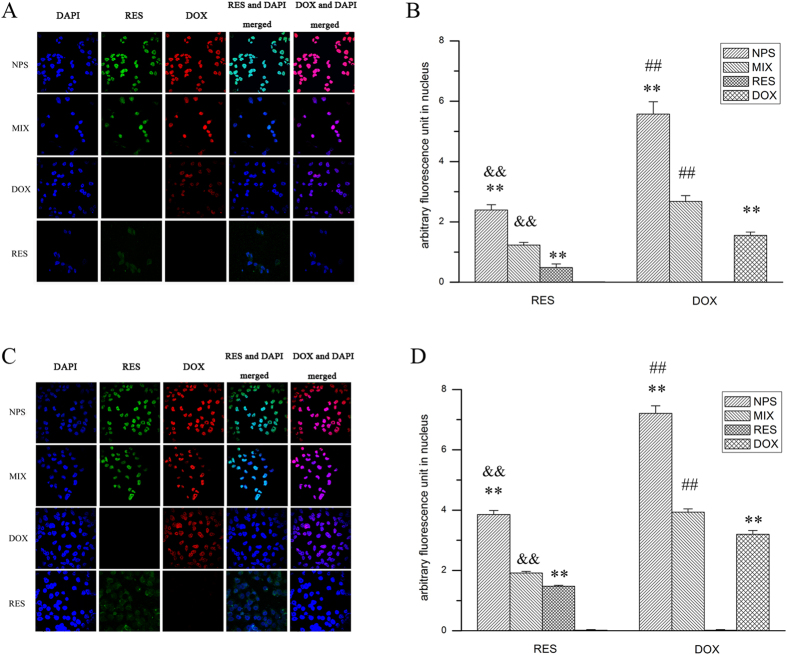
The cellular uptake of DOX and RES after cells were incubated with free DOX, free RES, MIX and DOX/RES-loaded NPS at 37 °C for 4 h in MCF-7/ADR cells (panel A) and MDA-MB-231/ADR cells (panel B). 60 × oil immersion objective and 10 × ocular lens. Data are mean ± SD. n = 3. *p < 0.05, **p < 0.01, vs same concentration of MIX. ^#^p < 0.05, ^##^p < 0.01, vs same concentration of free DOX. ^&^p < 0.05, ^&&^p < 0.01, vs same concentration of RES.

As showed in Fig. 3B, when free DOX was cultured with MDA-MB-231/ADR cells, considerable amount of DOX red fluorescence localized in the cytoplasm. When free RES was cultured with MDA-MB-231/ADR cells, a little amount of RES green fluorescence accumulated in the cell. Furthermore, when DOX/RES-loaded NPS was cultured with MDA-MB-231/ADR cells, a large amount of DOX red fluorescence and RES green fluorescence were localized in the nucleus, which led to a significant cytotoxicity of DOX/RES-loaded NPS on MDA-MB-231/ADR cells. The similar results were observed in MCF-7/ADR cells. Compared with MCF-7/ADR cells, DOX/RES-loaded NPS delivered more DOX and RES into the nucleus of MDA-MB-231/ADR cells.

### The effect of DOX/RES-loaded NPS on cell cycle in MDA-MB-231/ADR cells and MCF-7/ADR cells

**Figure 4:**
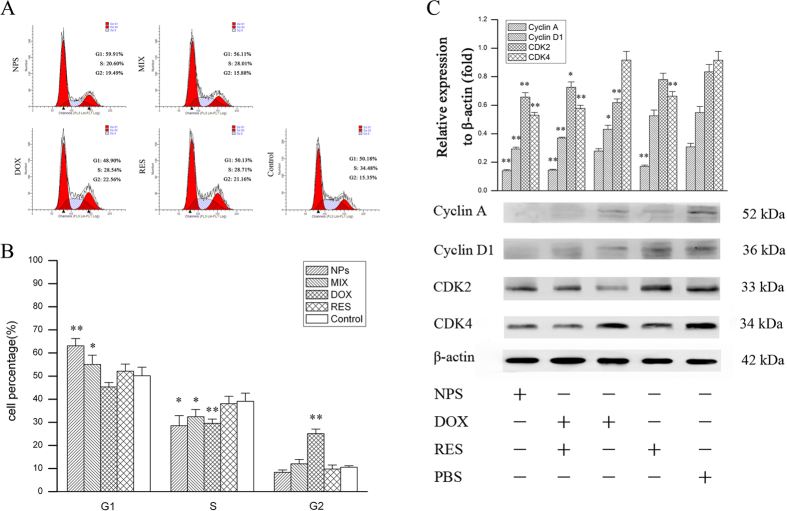
Cell cycle analysis after MDA-MB-231/ADR cells were cultured with free DOX, free RES, MIX and DOX/RES-loaded NPS for 24 h. Panel (A) is the typical pictures of flow cytometry. Panel (B) is the statistic results of cell cycle. Panel (C) is the western blot analysis of cell cycle-related protein expression. Data are mean ± SD. n = 3, *p < 0.05, **p < 0.01, vs control or PBS.

As showed in Fig. 4, free RES (60 μg/mL) showed none specific effect on cell cycle in MDA-MB-231/ADR cells. Free DOX (20 μg/mL) increased the percentage of cell in G2 phase in MDA-MB-231/ADR cells. The equivalent dose of mixture of free DOX and free RES exhibited G1 phase arrest in MDA-MB-231/ADR cells. In addition, the equivalent dose of DOX/RES-loaded NPS significantly exhibited G1 phase arrest in MDA-MB-231/ADR cells.

Cell cycle progression is positively regulated by the cyclins and cyclin-dependent kinases (CDKs)^27^. In order to investigated the mechanism that DOX/RES-loaded NPS arrested the cell cycle in G1 phase, the cell cycle related protein was determined by west blot, and the results are showed in Fig. 4C. DOX/RES-loaded NPS significantly reduced the expression of cyclin A1, cyclin D1, CDK2 and CDK4 in MDA-MB-231/ADR cells, which resulted in a G1 phase arrest. The mixture of DOX and RES showed the same effect as DOX/RES-loaded NPS. In addition, free DOX remarkably reduced the expression of cyclin D1 and CDK2 in MDA-MB-231/ADR cells. RES significantly reduced the expression of cyclin A1 and CDK4 in MDA-MB-231/ADR cells.

The effect of DOX/RES-loaded NPS on the cell cycle and cell cycle-related protein in MCF-7/ADR cells is showed in Supplementary Figure 5. The results exhibited the same tendency as in MDA-MB-231/ADR cells.

### The effect of DOX/RES-loaded NPS on apoptosis-related protein in MDA-MB-231/ADR cells and MCF-7/ADR cells

**Figure 5:**
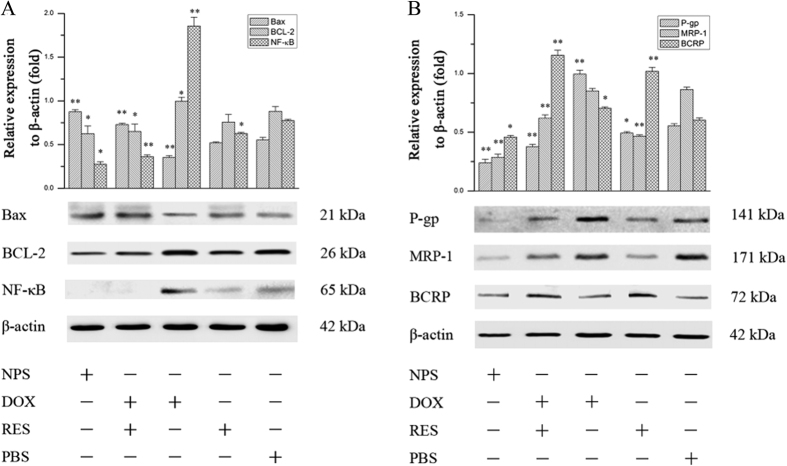
Western blot analysis of apoptosis-related proteins expression (panel A) and drug resistance-related proteins expression (panel B) in MDA-MB-231/ADR cells after cells were treated with free DOX, free RES, MIX and DOX/RES-loaded NPS for 24 h. Data are mean ± SD. n = 3, *p < 0.05, **p < 0.01, vs PBS.

**Figure 6:**
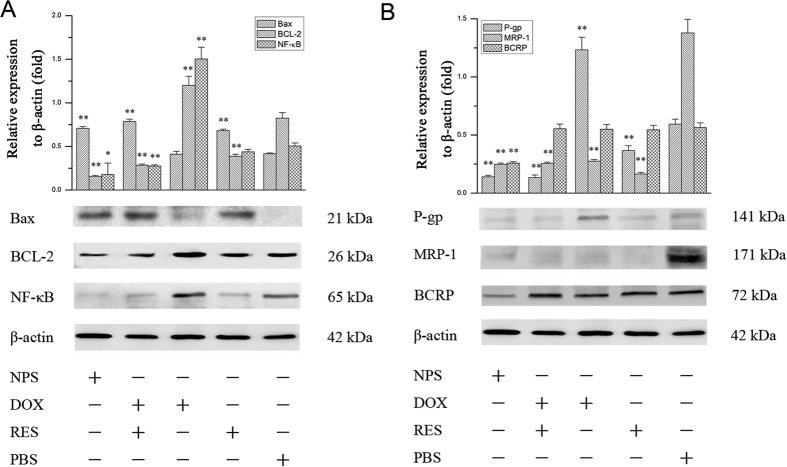
Western blot analysis of apoptosis-related proteins expression (panel A) and drug resistance-related proteins expression (panel B) in MCF-7/ADR cells after cells were treated with free DOX, free RES, MIX and DOX/RES-loaded NPS for 24 h. Data are mean ± SD, n = 3, *p < 0.05, **p < 0.01, vs PBS.

As showed in Fig. 5A, free DOX (20 μg/mL) obviously increased the expression of NF-κB in MDA-MB-231/ADR cells. However, free RES significantly decreased the expression of NF-κB in MDA-MB-231/ADR cells. Besides, free DOX decreased the expression of Bax and showed no effect on expression of BCL-2 in MDA-MB-231/ADR cells. Free RES (60 μg/mL) showed no effect on the expression of BCL-2 and Bax in MDA-MB-231/ADR cells. The mixture of DOX and RES obviously increased the expression of Bax and decreased the expression of NF-κB and BCL-2. Compared with free RES, the mixture of DOX and RES showed the stronger effect on NF-κB/BCL-2/Bax signal pathway. DOX/RES-loaded NPS (the equivalent concentration of RES and DOX was 60 μg/mL and 20 μg/mL, respectively) also significantly inhibited the expression of NF-κB and BCL-2 and increased the expression of Bax in MDA-MB-231/ADR cells. The effect of DOX/RES-loaded NPS on apoptosis-related protein in MCF-7/ADR cells is showed Fig. 6A, and the results were similar with in MDA-MB-231/ADR cells.

### The effect of DOX/RES-loaded NPS on drug resistance-related proteins in MDA-MB-231/ADR cells and MCF-7/ADR cells

The effect of DOX/RES-loaded NPS on P-gp, MRP-1 and BCRP in MDA-MB-231/ADR cells was investigated, and results are showed in Fig. 5B. Free DOX (20 μg/mL) obviously up-regulated the expression of P-gp and BCRP in MDA-MB-231/ADR cells. Free RES (60 μg/mL) significantly up-regulated the expression of BCRP and down-regulated the expression of P-gp in MDA-MB-231/ADR cells. Besides, free RES obviously down-regulated the expression of MRP-1 in MDA-MB-231/ADR cells. The mixture of DOX and RES significantly decreased the expressions of P-gp and MRP-1 in MDA-MB-231/ADR cells. The mixture of DOX and RES obviously up-regulated the expression of BCRP in MDA-MB-231/ADR cells, and it did not affect on the expression of MRP-1 in MCF-7/ADR cells. Moreover, the expression of MRP-1, P-gp and BCRP in MDA-MB-231/ADR cells was remarkably down-regulated by DOX/RES-loaded NPS (the equivalent concentration of RES and DOX was 60 μg/mL and 20 μg/mL, respectively). The effect of DOX/RES-loaded NPS on drug resistance-related protein in MCF-7/ADR cells is showed Fig. 6B, and the results were similar with in MDA-MB-231/ADR cells.

### *In vivo* anti-tumor activity of DOX/RES-loaded NPS

**Figure 7:**
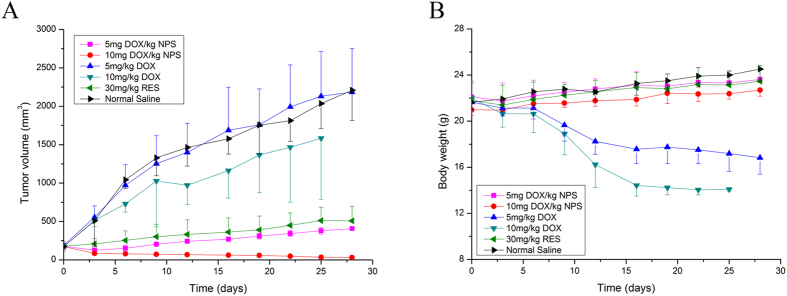
The tumor volume (panel A) and body weight (panel B) of tumor-bearing mice during period of treatment. Data are mean ± SD, n = 5.

The *in vivo* antitumor activities of DOX/RES-loaded NPS are showed in Fig. 7. The tumor volume increased fast when tumor-bearing mice were treated with normal saline. Meanwhile, after tumor-bearing mice were treated with 5 mg/kg free DOX, the tumor volume did not decrease as compared with normal saline treated tumor-bearing mice. 10 mg/kg free DOX significantly decreased the tumor volume. Besides, DOX/RES-loaded NPS obviously inhibited tumor growth in dose-dependent manner. In addition, as showed in Fig. 7B, the body weight significantly decreased in free DOX-treated tumor-bearing mice, while DOX/RES-loaded NPS did not cause significant body weight loss. Moreover, in 25 days, all tumor-bearing mice died in 10 mg DOX/kg treated group.

### Drug resistance-related protein and apoptosis-related proteins expressed in tumor tissue

**Figure 8:**
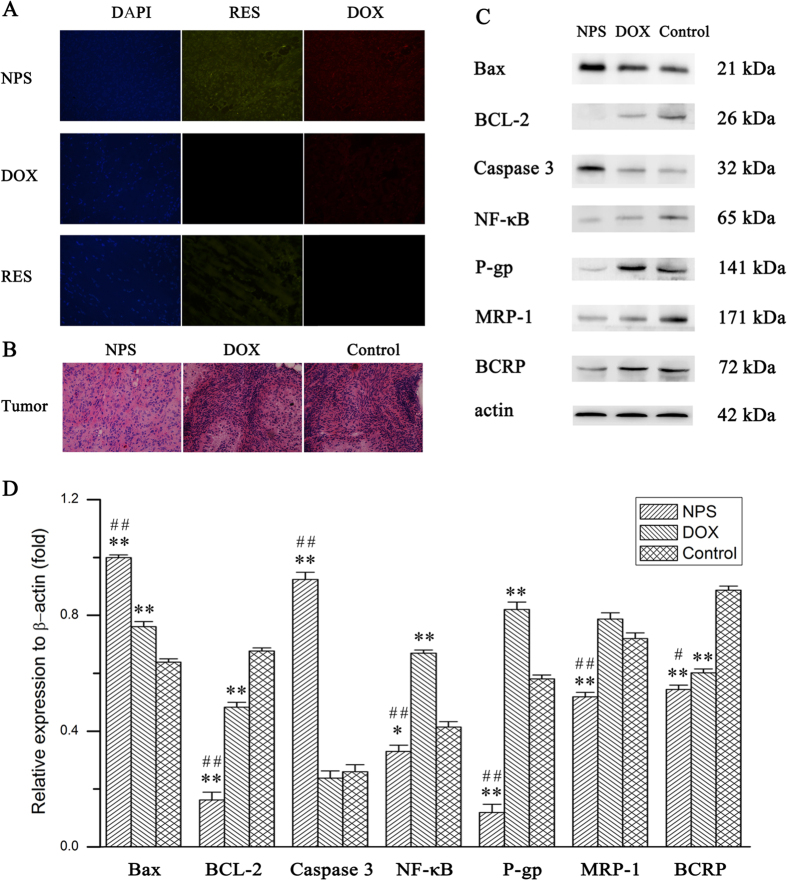
Panel (A) is the distribution of DOX and RES in section of tumor tissue at 24 h after treatment. 20 × oil immersion objective and 10 × ocular lens. Panel (B) is the H&E staining sections of tumor tissue from tumor-bearing nude mice treated with DOX/RES-loaded NPS (10 mg DOX/kg, 30 mg RES/kg), free DOX (10 mg/kg) and normal saline. 20 × oil immersion objective and 10 × ocular lens. Panel (C) is the apoptosis-related protein and drug resistance-related protein expressed in tumor tissue. Panel (D) is the statistic results of the apoptosis-related protein and drug resistance-related protein expressed in tumor tissue. Data are mean ± SD. n = 3. *p < 0.05, **p < 0.01, vs control; ^#^p < 0.05, ^##^p < 0.01, vs DOX.

After tumor-bearing mice were treated with DOX/RES-loaded NPS (10 mg DOX/kg), the expression of apoptosis-related protein and drug resistance-related protein in tumor tissue were determined by western blot. The results are showed in Fig. 8C,D. Compared with the tumor tissue from normal saline treated mice, the expression of Bax and caspase-3 were obviously up-regulated, and the expression of p-gp, MRP-1, BCRP, NF-κB and BCL-2 was remarkably down-regulated in the tumor tissue from DOX/RES-loaded NPS treated tumor-bearing mice. Free DOX (10 mg/kg) significantly dereased the protein expression of BCL-2 and BCRP, while markedly increased the protein expression of Bax, p-gp and NF-κB as compared with control group.

### The distribution of DOX and RES in tumor-bearing mice

**Figure 9:**
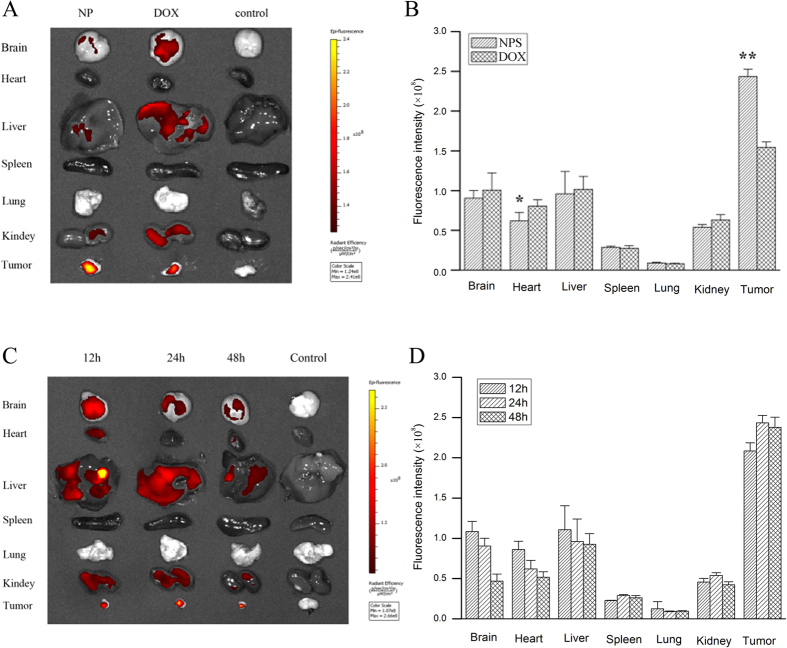
Panel (A) is living image pictures of DOX distribution at 24 h after DOX/RES-loaded NPS (10 mg DOX/kg, 30 mg RES/kg) and free DOX (10 mg/kg) were administered by tailvein injection. Panel (B) is the quantitative analysis of DOX distribution after DOX/RES-loaded NPS and free DOX were administered by tail vein injection in 24 h. Panel (C) is living image pictures of DOX distribution at 12 h, 24 h and 48 h after DOX/RES-loaded NPS were administered by tailvein injection. Panel (D) is the quantitative analysis of DOX distribution after NPS were administered by tail vein injection in 12 h, 24 h and 48 h. Data are mean ± SD. n = 3, *p < 0.05, **p < 0.01, vs free DOX.

**Figure 10:**
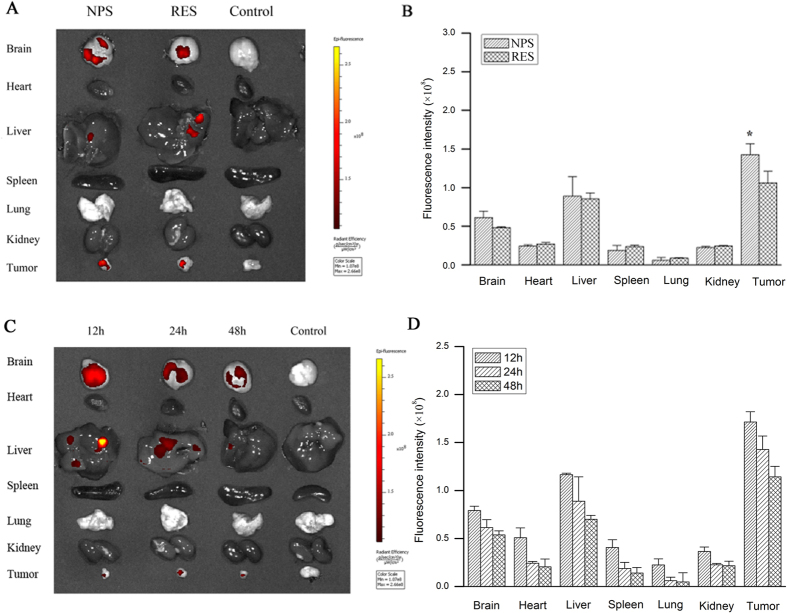
Panel (A) is living image pictures of RES distribution at 24 h after DOX/RES-loaded NPS (10 mg DOX/kg, 30 mg RES/kg) and free RES (30 mg/kg) were administered by tailvein injection. Panel (B) is the quantitative analysis of RES distribution after NPS and free RES were administered by tail vein injection in 24 h. Panel (C) is living image pictures of RES distribution at 12 h, 24 h and 48 h after NPS were administered by tailvein injection. Panel (D) is the quantitative analysis of RES distribution after DOX/RES-loaded NPS were administered by tail vein injection in 12 h, 24 h and 48 h. Data are mean ± SD. n = 3, *p < 0.05, vs free RES.

The distribution of DOX and RES in tumor-bearing mice is showed in Figs 9 and 10. DOX and RES were mainly distributed in the brain, liver, and kidney, only some amount of DOX and RES was distributed in tumor tissue after free DOX (10 mg/kg) or free RES (30 mg/kg) were respectively administered to tumor-bearing mice by tail vein. However, a large amount of DOX and RES distributed in tumor tissue, while only a little amount of DOX and RES were distributed in normal tissues after DOX/RES-loaded NPS (10 mg DOX/kg, 30 mg RES/kg) were administered. Compared with free DOX, DOX/RES-loaded NPS significantly reduced the distribution of DOX in heart tissue. Besides, a relative high level of DOX and RES in tumor tissue was maintained for a longer time after DOX/RES-loaded NPS was administered.

As showed in Fig. 8A, after free DOX (10 mg/kg) and free RES (30 mg/kg) were respectively administered to tumor-bearing mice by tail vein, only a little amount of DOX and RES were distributed in the section of tumor tissue. However, both DOX and RES were extensively distributed in the section of tumor tissue after DOX/RES-loaded NPS (10 mg DOX/kg) was administered.

### H&E staining analysis

**Figure 11:**
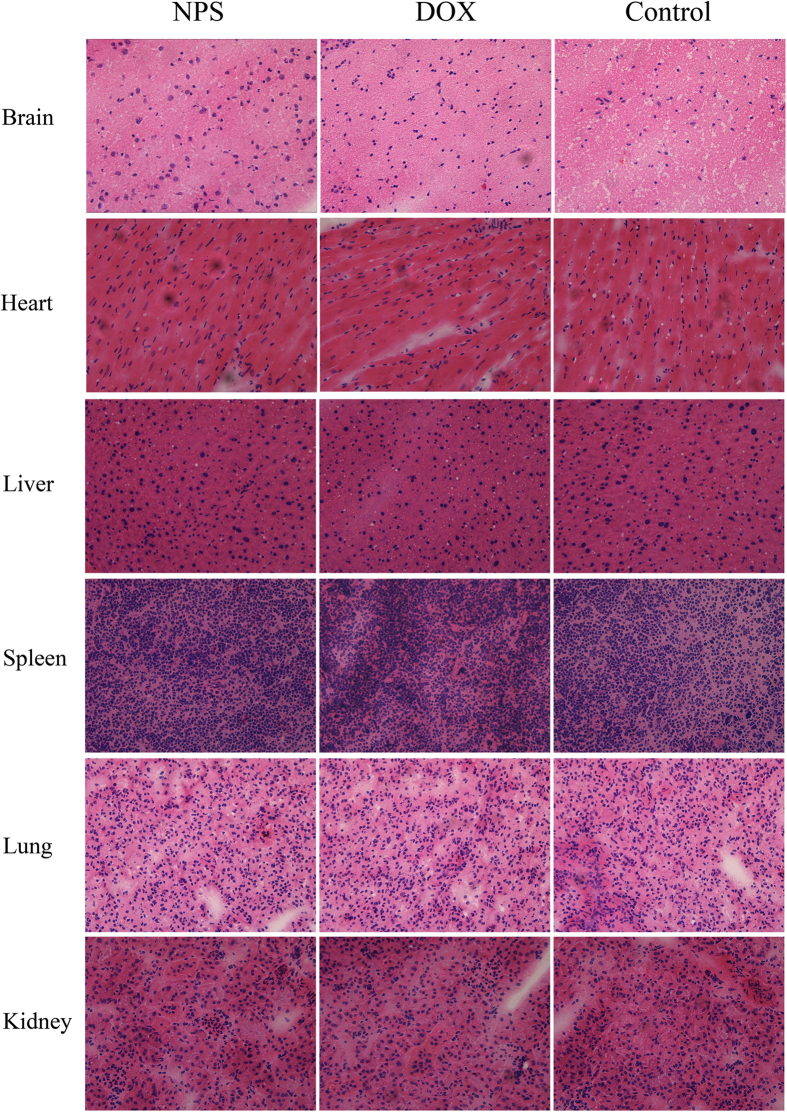
Representative H&E staining sections of the brain, heart, liver, spleen, lung and kidney from tumor-bearing mice treated with NPS (10 mg DOX/kg, 30 mg RES/kg), free DOX (10 mg/kg) and normal saline. 20 × oil immersion objective and 10 × ocular lens.

After tumor-bearing mice had been treated with DOX/RES-loaded NPS (10 mg DOX/kg), the H&E staining sections of heart, liver, spleen, lung, and kidney from tumor-bearing nude mice are showed in Fig. 11. No obvious histopathological changes were observed in heart, kidney, liver, spleen, and lung section from DOX/RES-loaded NPS treated tumor-bearing nude mice. Nevertheless, the cardiac section from DOX-treated (10 mg/kg) tumor-bearing nude mice showed marked neutrophils infiltration. Also, as shown in Fig. 8B, obviously histopathological changes were observed in tumor tissue section from DOX/RES-loaded NPS (10 mg DOX/kg) treated tumor-bearing mice.

## Discussion

There are four release mechanisms for PLGA nanoparticle to release drug: (1) diffusion via water-filled pores in PLGA nanoparticle, (2) diffusion through PLGA polymer, (3) osmotic pressure in PLGA nanoparticle, and (4) degradation of PLGA^28^. The degradation of PLGA mainly occurs by ester bond broken in the polymer backbone between glycolic acid and lactic acid. In fact, the degradation of PLGA *in vitro* and *in vivo* is very slow^29^,^30^. Thus, diffusion via water-filled pores is the most common mechanism for PLGA nanoparticle to release drug^28^.

DOX/RES-loaded NPS exhibited a biphasic drug release pattern in pH 7.4, pH 6.4 and pH 5.0 medium. The initial burst release was mainly due to the release of DOX and RES encapsulated in outer lipid layer of NPS. The initial burst release followed a sustained release process. The sustained release of DOX and RES from NPS was attributed to diffusion-mediated release from nanoparticles and the gradual degradation and corrosion of PLGA matrix^31^. Furthermore, the faster release of DOX and RES from NPS in pH 5 medium was resulted from the broken of hydrazone linkage between the CHO and PEG. This characteristic led to the burst release of DOX and RES in the lysosome of tumor cells.

The caspase-3 activity induced by DOX/RES-loaded NPS in MDA-MB-231/ADR cells and MCF-7/ADR cells was determined. The results indicated that the DOX/RES-loaded NPS induced greater caspase-3 activity than mixture of free DOX and free RES, free DOX and free RES on MDA-MB-231/ADR cells and MCF-7/ADR cells. The mixture of free DOX and free RES induced higher caspase-3 activity than free DOX and free RES on MDA-MB-231/ADR cells and MCF-7/ADR cells. Besides, DOX/RES-loaded NPS exhibited greater cytotoxicity than free DOX and free RES on MDA-MB-231/ADR cells and MCF-7/ADR cells. The cytotoxicity of a mixture of free DOX and free RES was higher than free DOX and free RES. The above results indicated that the effect of DOX/RES-loaded NPS on caspase-3 activity in tumor cells was well consistent with the cytotoxicity of DOX/RES-loaded NPS on MDA-MB-231/ADR cells and MCF-7/ADR cells. These results also indicated that RES played a crucial role in overcoming DOX resistance and enhancing the cytotoxicity of DOX on MDA-MB-231/ADR cells and MCF-7/ADR cells.

Because the main action target of DOX is nuclear DNA, thus the cellular uptake and intracellular localization of DOX and RES delivered by DOX/RES-loaded NPS were observed by confocal laser scanning microscopy (CLSM). The results indicated that RES increased the cellular accumulation of DOX, and DOX/RES-loaded NPS enhanced the accumulation of DOX and RES in the nucleus. In addition, compared with free RES, the cellular uptake of RES was greater in the mixture of DOX and RES group. This phenonema is interesting, and the exact mechanism is worth to further investigate. Moreover, compared with MCF-7/ADR cells, DOX/RES-loaded NPS delivered more DOX and RES to the nucleus of MDA-MB-231/ADR cells. Aydar E. found that MDA-MB-231 cells expressed much more sigma receptor as compared with MCF-7 cells^24^. Because DOX/RES-loaded NPS was modified with a ligand of Sigma receptor, it was easy for DOX/RES-loaded NPS to bind itself with sigma receptor over-expressed tumor cells and delivered much amount of DOX and RES into MDA-MB-231/ADR cells^32^. Consequently, DOX/RES-loaded NPS showed greater cytotoxicity on MDA-MB-231/ADR cells than on MCF-7/ADR cells.

When DNA is damaged, the cell cycle is stagnated at G1 phase^33^,^34^. The experimental results indicated that DOX/RES-loaded NPS arrested the cell cycle in G1 phase, while free DOX arrested the cell cycle in G2 phase in MDA-MB-231/ADR cells and MCF-7/ADR cells. Cell cycle progression is positively regulated by the cyclins and cyclin-dependent kinases. Cyclin A plays a crucial role in both S phase and G2/M transition in the cell cycle. Cyclin D1 regulates the progression of G1 to S phase. The cyclin-CDK complexes are involved in different periods of the cell cycle. The formation of cyclin A and CDK2 dimer complex initiates S phase. The formation of cyclin D and CDK4 complex promotes cell through G1/S checkpoint to S phase^35^. In order to investigated the mechanism that DOX/RES-loaded NPS arrested the cell cycle in G1 phase, the cell cycle-related protein was determined by western blot. The experimental results indicated that through inhibiting the formation of cyclin A and CDK2 dimers complex as well as cyclin D and CDK4 complex, DOX/RES-loaded NPS induced G1 phase arrest in MDA-MB-231/ADR cells and MCF-7/ADR cells.

It was reported that NF-κB stimulated the proliferation and inhibited the apoptosis of human breast cancer cells^36^. Activation of NF-κB led to autoimmune disease and cancer. In mammals, Bcl-2 is called as an anti-apoptosis gene, which can suppress cell apoptosis and protects cell from various cytotoxic attack. BAX is called as a pro-apoptosis gene, which can promote cell apoptosis. The ratio between BCL-2 and Bax determines survival or death after cells are stimulated with an apoptotic signal^37^. BCL-2 is regulated by NF-κB-dependent transcription^38^. This indicates NF-κB/BCL-2/Bax signal pathway is involved in regulating cell proliferation, differentiation, and apoptosis^25^,^39^,^40^. Therefore, the effects of DOX/RES-loaded NPS on the NF-κB/BCL-2/Bax signal pathway were investigated. The results indicated that DOX/RES-loaded NPS significantly inhibited the expression of NF-κB and BCL-2, and increased the expression of Bax in MDA-MB-231/ADR cells and MCF-7/ADR cells. This implied that when RES and DOX were co-delivered into tumor cells by DOX/RES-loaded NPS, the expression of NF-κB was down-regulated. Subsequently, the ratio between BCL-2 and Bax was decreased, and the activity of caspase-3 was increased, which led to the apoptosis of DOX-resistant tumor cells.

Furthermore, the recent work has showed that NF-κB also involves in drug resistance. Up-regulation of NF-κB results in drug resistance through increasing the expression of ABCs multidrug transport protein in human cancer cells^41^,^42^,^43^,^44^,^45^. The western blot results indicated that DOX/RES-loaded NPS significantly inhibited the expression of NF-κB and ABC multidrug transport proteins such as MRP-1, P-gp and BCRP in MDA-MB-231/ADR cells and MCF-7/ADR cells. Therefore, the efflux of DOX from DOX-resistant tumor cells was blocked by using DOX/RES-loaded NPS. Consequently, DOX resistance in MDA-MB-231/ADR cells and MCF-7/ADR cells was reversed, and the antitumor activity was enhanced.

The nonselective distribution of DOX in the normal organs results in the serious systemic toxicity, which limits the clinic application of DOX. The *in vivo* experiments indicated DOX/RES-loaded NPS could simultaneously deliver DOX and RES to tumor tissue and increased the accumulation of DOX and RES in tumor tissue. Meanwhile, the distribution of DOX and RES in normal tissue was obviously reduced. Compared with free DOX, DOX/RES-loaded NPS obviously decreased the distribution of DOX in heart tissue. Consequently, DOX/RES-loaded NPS decreased cardiotoxicity of DOX. However, free DOX treatment caused obviously histopathological changes in cardiac tissue and showed marked neutrophils infiltration in cardiac section.

In addition, the protein expression of NF-κB, BCL-2, MRP-1, P-gp, and BCRP were down-regulated, and expression of caspase-3 and Bax were up-regulated in tumor tissue by using DOX/RES-loaded NPS. Those results implied that DOX/RES-loaded NPS increased the accumulation of DOX and RES in DOX-resistant tumor cells by down-regulating of p-gp, MRP-1 and BCRP expression *in vivo*. At the same time, DOX/RES-loaded NPS induced the tumor cells aptotosis by decreasing BCL-2 expression and increasing Bax and caspase-3 expression *in vivo*. Eventually, the DOX resistance was overcome, and the antitumor activity was enhanced by using DOX/RES-loaded NPS.

In a word, DOX/RES-loaded NPS could overcome the DOX resistance and had the potential in the treatment of DOX-resistant breast cancer.

## Additional Information

**How to cite this article**: Zhao, Y. *et al*. Doxorubicin and resveratrol co-delivery nanoparticle to overcome doxorubicin resistance. *Sci. Rep.* **6**, 35267; doi: 10.1038/srep35267 (2016).

## Supplementary Material

Supplementary Information
