# Supplementary material for: Doxorubicin and resveratrol co-delivery nanoparticle to overcome doxorubicin resistance
Source: Sci Rep. 2016 Oct 12;6:35267. doi: 10.1038/srep35267 (PMC5059704; doi:10.1038/srep35267)

Supplementary Information

Doxorubicin and resveratrol co-delivery nanoparticle to overcome doxorubicin resistance

Yuan Zhao[1], Meng-lei Huan[1#], Miao Liu[1#], Ying Cheng[1], Yang Sun[2], Han Cui[1], Dao-zhou Liu[1], Qi-bing Mei[2], Si-yuan Zhou[1, 2] *

[1]Department of Pharmaceutics, School of Pharmacy, Fourth Military Medical University, Xi'an, 710032, China.

[2]Key laboratory of gastrointestinal pharmacology of Chinese medicine, Fourth Military Medical University, Xi'an, 710032, China.

*Corresponding author; [#]The author contributed equally to this work.

Running title: co-delivery of doxorubicin and resveratrol.

Corresponding author: Si-yuan Zhou.

Tel: 86 29 84776783.

Fax: +86 29 84779212.

E-mail: zhousy@fmmu.edu.cn

Mail address: Changle West Road 17, Shaanxi province, Xi'an, 710032, China.

Conflicts of Interest/Disclosures: None

Supplementary figure 1. Synthetic route of CHO-hyd-PEG-AA.

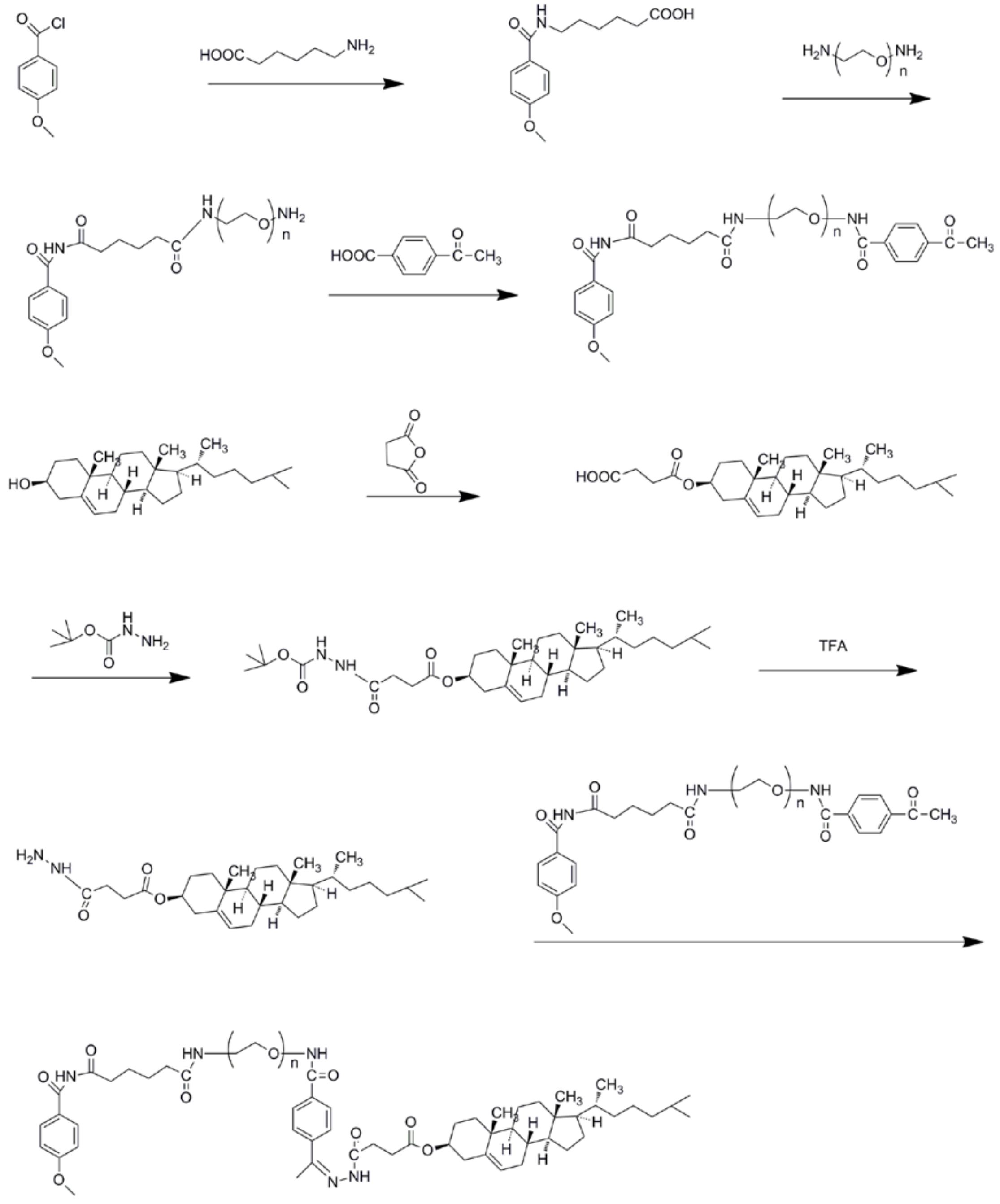

Supplementary figure 2. The ^{1}H NMR spectrum (dissolved in $CHCl_3$) of CHO-hyd-PEG-AA.

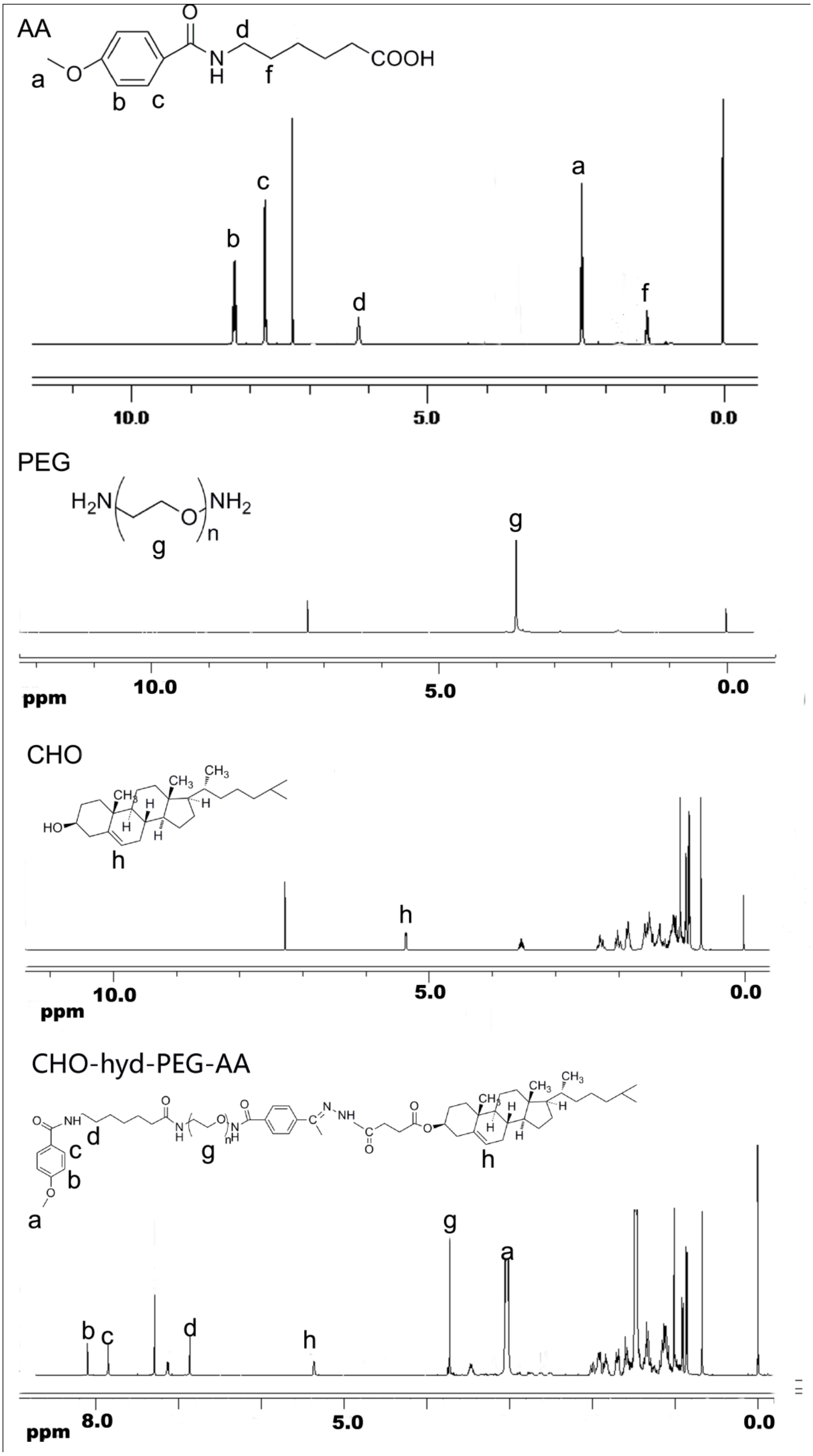

Supplementary figure 3. The FTIR spectrum of CHO-hyd-PEG-AA.

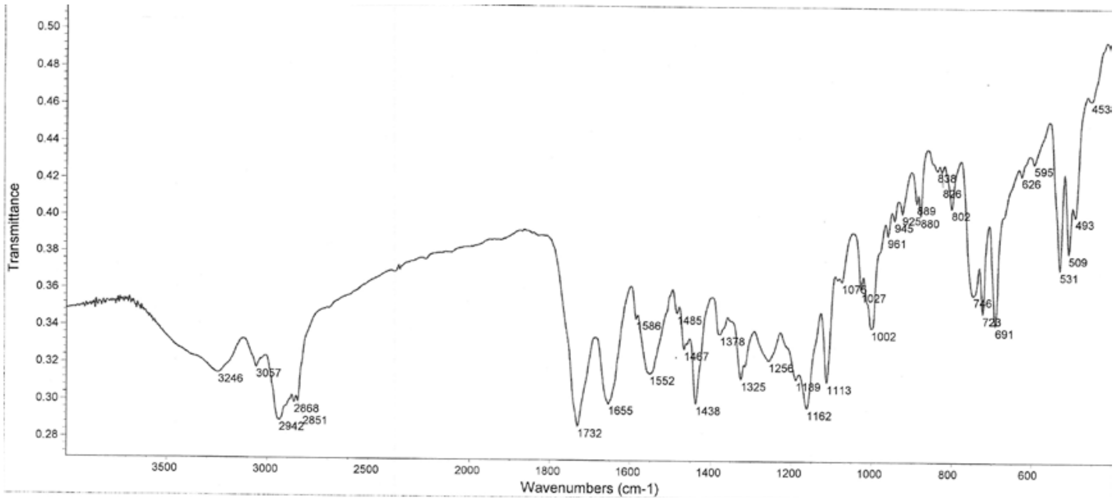

Supplementary figure 4. Cytotoxicity of blank nanoparticle on MDA-MB-231/ADR cells (A) and MCF-7/ADR cells (B).

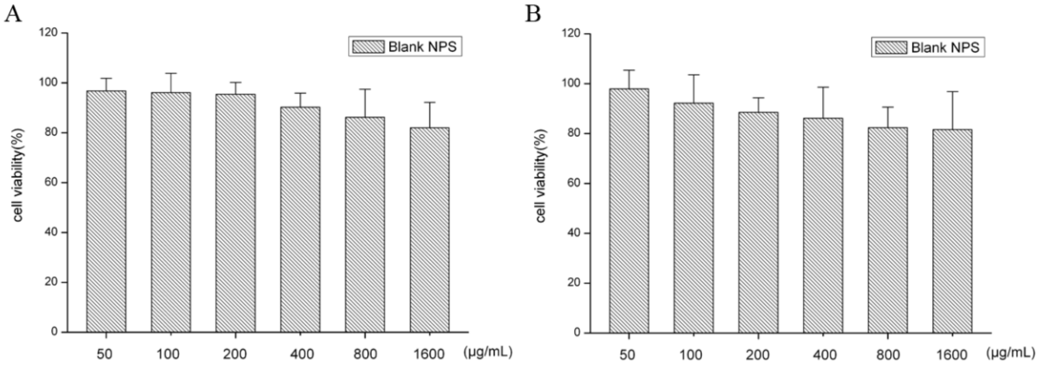

Supplementary figure 5. Cell cycle analysis after MCF-7/ADR cells were cultured with free DOX, free RES, MIX and DOX/RES-loaded NPS for 24 h. Panel A is the typical pictures of flow cytometry in MCF-7/ADR cells. Panel B is the statistic results of cell cycle. Panel C is the western blot analysis of cell cycle-related proteins expression. Data are mean±SD, n=3, *p<0.05, **p<0.01, vs control or PBS.

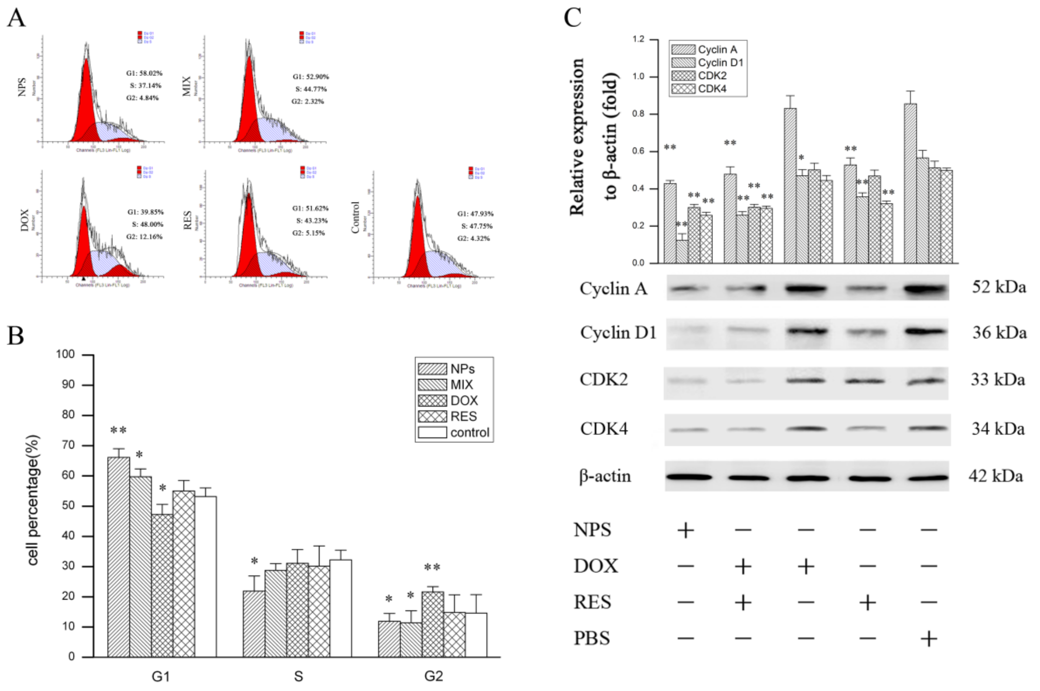

Supplement: Supplementary Information [file srep35267-s1.pdf]
